# Psychosoziale Belastung und psychosoziale Unterstützung für Fachkräfte im Gesundheitswesen während der COVID-19-Pandemie: Was haben Mitarbeiter*innen einer Universitätsklinik als hilfreich erlebt?

**DOI:** 10.1007/s00278-022-00642-6

**Published:** 2023-01-26

**Authors:** Julia Holl, Anna Berning, Manuel Joachim Benetik, Marie Ottilie Frenkel, Annette Bellm, Beate Ditzen, Kirsten Bikowski, Katharina Konrath, Grit Parker, Jannik Porath, Franziska Zumbaum-Fischer, Svenja Taubner

**Affiliations:** 1 Institut für Psychosoziale Prävention, Zentrum für Psychosoziale Medizin, Universitätsklinikum Heidelberg, Heidelberg, Deutschland; 2 Institut für Sport und Sportwissenschaft, Fakultät für Verhaltens- und Kulturwissenschaften, Ruprecht-Karls-Universität Heidelberg, Heidelberg, Deutschland; 3 Institut für Medizinische Psychologie, Zentrum für Psychosoziale Medizin, Universitätsklinikum Heidelberg, Heidelberg, Deutschland; 4 Betriebliche Psychosoziale Beratung für Beschäftigte der Universitätsklinik und der Universität Heidelberg, Betriebsärztlicher Dienst, Universitätsklinikum Heidelberg, Heidelberg, Deutschland; 5 Stabsstelle Betriebliches Gesundheitsmanagement, Geschäftsbereich Personalmanagement, Universitätsklinikum Heidelberg, Heidelberg, Deutschland; 6 Betriebliche Sozial- und Suchtberatung, Stabsstelle Konflikt- und Krisenmanagement, Geschäftsbereich Personalmanagement, Universitätsklinikum Heidelberg, Heidelberg, Deutschland; 7 Klinik für Psychosomatische Medizin und Innere Medizin, Zentrum für Psychosoziale Medizin, Universitätsklinikum Heidelberg, Heidelberg, Deutschland

**Keywords:** Corona Pandemie, Depressivität, Klinikpersonal, Inanspruchnahme, Mixed-methods, Corona pandemic, Depression, Healthcare professionals, Utilization, Mixed-methods

## Abstract

**Hintergrund:**

Ausgangspunkt der Studie ist die hohe psychosoziale Belastung von Fachkräften im Gesundheitswesen bei gleichzeitig geringer Inanspruchnahme eines Beratungs- und Unterstützungsangebots für Mitarbeiter*innen einer Universitätsklinik während der COVID-19-Pandemie.

**Ziel der Arbeit:**

Die vorliegende Studie untersucht den Grad des psychosozialen Belastungserlebens von Fachkräften im Gesundheitswesen (*n* = 859) einer Universitätsklinik sowie Kenntnis, Inanspruchnahme und Bewertung innerbetrieblicher psychosozialer Versorgungsstrukturen.

**Material und Methoden:**

Im Rahmen einer Online-Befragung (21.07.–19.10.2021) wurden die subjektiv empfundene Belastung durch die COVID-19-Pandemie, Depressivität (PHQ-8) des Personals sowie Kenntnis, Inanspruchnahme und Bewertung möglicher Versorgungsangebote der Universitätsklinik erhoben. Zusätzlich wurden qualitative Daten zu Informationsquelle, -vermittlung sowie Art der Kontaktaufnahme der innerbetrieblichen Versorgungsstrukturen erhoben.

**Ergebnisse:**

Trotz hoher psychosozialer Belastung und überwiegender Kenntnis der Existenz innerbetrieblicher psychosozialer Versorgungsangebote ist deren Inanspruchnahme bei Fachkräften im Gesundheitswesen gering. Insbesondere der Kontakt zu Patient*innen stellte einen Risikofaktor für das psychosoziale Belastungserleben dar. Die qualitative Analyse zeigt, dass die Mitarbeiter*innen mehr über E‑Mails und Newsletters informiert werden möchten sowie eine übersichtliche Darstellung der Angebote vermissen.

**Diskussion:**

Eine Überarbeitung der Angebotsdarstellung und Kontaktwege gemäß den Wünschen des Personals könnte die Inanspruchnahme innerbetrieblicher Angebote verbessern.

## Abstract (en)

**Background:**

The starting point of the study is the high psychosocial distress of healthcare professionals and the low utilization of a counselling and support service specifically developed for employees of a university hospital during the COVID-19 pandemic.

**Objective:**

The present study examines the degree of psychosocial distress experienced as well as knowledge, utilization, and evaluation of in-house psychosocial care structures based on a large sample of healthcare professionals (*n* = 859) at a university hospital**.**

**Material and methods:**

Within the framework of an online survey (21 July 2021–19 October 2021), the subjectively perceived stress caused by the COVID-19 pandemic, depressive symptoms (PHQ-8) of the healthcare professionals as well as knowledge, utilization, and evaluation of possible care services offered by the university hospital were collected. In addition, qualitative data were collected on the source of knowledge, the provision of information and the type of contact with the in-house care structures.

**Results:**

Despite high psychosocial distress and predominant knowledge about the existence of in-house psychosocial care services, the utilization of these services is low among healthcare professionals. In particular, contact with patients was a risk factor for the psychosocial distress. The qualitative analysis shows that employees would prefer to be informed via email and newsletter and that they miss a clear presentation of care structures.

**Conclusion:**

A revision of the presentation of offers and contact channels according to the wishes of the staff could improve the acceptance of in-house services.

Fachkräfte im Gesundheitswesen sind durch die COVID-19-Pandemie enormen psychosozialen Belastungen ausgesetzt. Innerbetriebliche psychosoziale Versorgungsstrukturen erscheinen dringend notwendig, um die Fachkräfte beim Umgang mit der aktuellen Belastung zu unterstützen. Ein in der Universitätsklinik Heidelberg implementiertes psychosoziales Versorgungsangebot für Mitarbeitende zeigte jedoch entgegen der Erwartungen eine geringe Inanspruchnahme. Empirische Daten zu Kenntnis, Inanspruchnahme und Bewertung innerbetrieblicher psychosozialer Versorgungsstrukturen könnten dazu beitragen, deren Akzeptanz zu verbessern und die Inanspruchnahme zu steigern.

## Hintergrund und Fragestellung

Eine Vielzahl (inter-)nationaler Studien zeigt, dass Fachkräfte im Gesundheitswesen enormen psychosozialen Belastungen sowohl während als auch nach den COVID-19-Pandemie-Wellen ausgesetzt waren (u. a. Batra et al. 2020; Bohlken et al. 2020; Mulfinger et al. 2020; Osório et al. 2022; Pappa et al. 2020; Van Wert et al. 2022; Wu et al. 2021). In der ersten Welle der COVID-19-Pandemie dominierten Stressoren wie die Angst vor Ansteckung, Unsicherheit und mangelndes Wissen sowie Sorgen um das Team (Frenkel et al. 2022). Darüber hinaus ist bei Fachkräften des Gesundheitswesens eine pandemiebedingt erhöhte psychopathologische Belastung gut dokumentiert (u. a. Aymerich et al. 2022; Dragioti et al. 2022; Hill et al. 2022; Krishnamoorthy et al. 2020; Marvaldi et al. 2021; Riedel-Heller und Bohlken 2020; Saragih et al. 2021). Besonders hervorzuheben ist das aktuelle Umbrella-Review von Chutiyami et al. (2022), in das 40 systematische Reviews mit einer Gesamtstichprobe von *n* = 3.245.768 einbezogen wurden. Als die vorherrschenden pandemiebedingten Symptome psychischer Belastung bei Beschäftigten im Gesundheitswesen wurden Ängste, Depressionen und Stress/posttraumatische Belastungsstörung identifiziert. Weibliches Geschlecht, junges Alter und direkter Kontakt zu COVID-19-Patient*innen wurden sowohl in dem Umbrella-Review (Chutiyami et al. 2022) als auch in einem Großteil anderer Untersuchungen (Bohlken et al. 2020; Cai et al. 2020; Hao et al. 2021; Hill et al. 2022; Manchia et al. 2022; Mulfinger et al. 2020; Muller et al. 2020) als bedeutsame Risikofaktoren identifiziert. Daten chinesischer Studien wiesen bereits zu Beginn der COVID-19-Pandemie darauf hin, dass auch Personal, das nicht unmittelbar mit COVID-19-Patient*innen arbeitet, von psychosozialer Belastung betroffen ist (Li et al. 2020; Liang et al. 2020). Angesichts der hohen psychosozialen Belastung von Fachkräften des Gesundheitswesens während der COVID-19-Pandemie scheinen innerbetriebliche psychosoziale Unterstützungsangebote dringend notwendig zu sein, um den Umgang mit der aktuellen Belastung zu erleichtern.

Im Gegensatz zur Untersuchung der psychosozialen Belastung ist die empirische Datenlage zur Entwicklung, Implementation und Bewertung von innerbetrieblichen Versorgungsangeboten für Fachkräfte des Gesundheitswesens unterrepräsentiert (Branjerdporn et al. 2022). Das kürzlich erschienene Review von Branjerdporn et al. (2022) gibt, basierend auf einer Zusammenschau von 55 Studien mit unterschiedlichen methodischen Ansätzen (beispielsweise Kommentare, Kohortenstudien, qualitativen Studien), einen Überblick zu den bislang international entwickelten innerbetrieblichen Maßnahmen zur Förderung der psychischen Gesundheit im Rahmen von Pandemien (ausgelöst durch Infektionen mit dem Severe Acute Respiratory Syndrome Coronoavirus [SARS-CoV] und COVID-19). Darunter finden sich Initiativen am Arbeitsplatz (z. B. Ruheräume, kostenlose Mahlzeiten), Selbsthilfe- und Resilienzgruppen für das Personal, Telefon-Hotlines und individuelle Programme zur Förderung der psychischen Gesundheit (z. B. psychologische Erste Hilfe sowie psychotherapeutische/psychiatrische Intervention). Als wesentliche Einschränkung wurde das Fehlen einer empirischen Programmbewertung angesichts der Mehrheit nichtempirischer Kommentare festgestellt.

### Niedrigschwelliges psychosoziales Beratungs- und Unterstützungsangebot

#### Entwicklung und Implementation

Bereits zu Beginn der Pandemie im März 2020 wurde unter der Leitung der Institute für Medizinische Psychologie und Psychosoziale Prävention ein niedrigschwelliges Beratungs- und Unterstützungsangebot zur psychosozialen Unterstützung für das Personal der Universitätsklinik Heidelberg initiiert. Das klinik- und institutsübergreifende Angebot war mit den erfahrenen Mitarbeiter*innen des Zentrums für Psychosoziale Medizin, der betrieblichen Sozial- und Suchtberatung, dem betrieblichen Gesundheitsmanagements sowie der betrieblichen psychosozialen Beratung für Beschäftigte der Universitätsklinik und der Universität Heidelberg, die über die notwendige Fachkompetenz und Beratungserfahrung verfügten, interdisziplinär ausgerichtet. Das Angebot wurde auf der Homepage des Zentrums für Psychosoziale Medizin der Universitätsklinik, in den regelmäßigen TaskForce-Newslettern *SARS-CoV-2/COVID-19* sowie per Flyer in verschiedenen Einrichtungen der Universitätsklinik (u. a. medizinische Stationen sowie Aufenthaltsräume) beworben. Die Kontaktaufnahme erfolgte in der Anfangszeit telefonisch oder per E‑Mail mit 2 Diensten. Die Mitarbeiter*innen des Angebots führten diese Tätigkeit ehrenamtlich aus. Durch das psychosoziale Hilfsangebot konnte das Personal der Universitätsklinik Heidelberg telefonisch oder per Videochat eine Beratung zu Entlastung, Unterstützung und Vermeidung von Belastungsfolgen erhalten. Im Bedarfsfall (beispielsweise auf Wunsch der Betroffenen) wurde ebenfalls eine Beratung außerhalb der Universitätsklinik vermittelt. Kontaktaufnahme und Beratung erfolgten vertraulich und unterlagen der Schweigepflicht. Darüber hinaus wurden auf der Homepage des Beratungs- und Unterstützungsangebotes psychoedukative Materialien u. a. zu psychischen Belastungsreaktionen auf pandemiebedingte Auswirkungen sowie Ressourcen zu Bewältigungsstrategien zur Verfügung gestellt.

#### Geringe Nachfrage des Angebots als Hintergrund einer empirischen Untersuchung

Obwohl die empirischen Daten darauf hindeuten, dass sich bei Fachkräften im Gesundheitswesen pandemiebedingte Auswirkungen auf das psychische Wohlbefinden zeigen (Chutiyami et al. 2022), wurde das psychosoziale Versorgungsangebot nicht so sehr in Anspruch genommen wie erwartet. Diese Diskrepanz bildete den Startpunkt für eine empirische Untersuchung der psychosozialen Belastung sowie des innerbetrieblichen psychosozialen Versorgungsbedarfs der Mitarbeiter*innen der Uniklinik in der andauernden COVID-19-Pandemie. Weiterhin sollten Informationen zu den bereits existierenden psychosozialen Versorgungsangeboten für das Personal der Uniklinik gesammelt werden, mit dem Ziel, daraus Schlussfolgerungen für zukünftige psychosoziale Versorgungsstrukturen ableiten zu können.

## Ziel der Arbeit

Aus der oben beschriebenen Situation ableitend ist es das Ziel der Arbeit, Prädiktoren für die aktuelle Belastung und die Inanspruchnahme von Hilfsangeboten bei Mitarbeiter*innen einer Universitätsklinik zu untersuchen. Aufgrund der besonderen Situation der COVID-19-Pandemie wurde die Studie explorativ konzipiert, wodurch erste Hinweise auf mögliche Zusammenhänge gesammelt und in weiteren Untersuchungen hypothesengeleitet überprüft werden können.

## Material und Methoden

### Studiendesign und Stichprobe

Die querschnittliche Studie wurde an der Universitätsklinik Heidelberg durchgeführt. Das Personal der Universitätsklinik umfasst 11.315 Mitarbeitende in 7 medizinischen Zentren (aufgeteilt in 50 medizinische Abteilungen) und 8 medizintheoretischen Instituten (Stand 04.11.2022; (Heidelberg 2022)). Die 5‑ bis 10-minütige, anonyme und freiwillige Erhebung erstreckte sich vom 21.07.2021 bis zum 19.10.2021. In diesem Erhebungszeitraum zeichnete sich eine langsame Steigung der deutschlandweiten Coronainzidenz ab, wobei diese sich relativ zu den vorherigen Pandemiewellen noch auf einem geringen Niveau befand (21.07.2021: 2203 Neuinfektionen, 20.10.2021: 17.015 Neuinfektionen; Statista 2022b). Analog dazu stieg in diesem Zeitraum die deutschlandweite Zahl der intensivmedizinischen Behandlungen deutlich an (20.07.2021: 361, 20.10.2021: 1482; Statista 2022a). Nach der Datenbereinigung enthielt der finale Datensatz 859 Personen (Alter: Median: 41 bis 45 Jahre, Range: 16 bis 70 Jahre; Geschlecht: weiblich: 649, männlich: 204, divers: 6). Ein positives Ethikvotum erfolgte durch die unabhängige Ethikkommission der Fakultät für Verhaltens- und Empirische Kulturwissenschaften der Universität Heidelberg (AZ Tau 2021 3/1).

### Datenerhebung

Die Studienteilnehmer*innen wurden über E‑Mail-Verteiler der Universitätsklinik, dem *SARS-CoV-2/COVID*-TaskForce-Newsletter sowie Intranet-Postings rekrutiert. Es wurden alle Mitarbeitenden der Universitätsklinik gebeten, sich an der Studie zu beteiligen. Es gab keine Ausschlusskriterien. Auf ein Mindestalter als Teilnahmevoraussetzung wurde verzichtet, da die vulnerable Gruppe der Auszubildenden ebenfalls in die Erhebung integriert werden sollte. Da aus den bisherigen empirischen Untersuchungen deutlich wurde, dass nicht nur Gesundheitsfachkräfte mit COVID-19-Patient*innenkontakt von psychosozialen Belastungen während der Pandemie betroffen sind (Li et al. 2020; Liang et al. 2020), wurden in der vorliegenden Arbeit alle Mitarbeitenden der Universitätsklinik Heidelberg und somit Personen mit Beschäftigungen im Gesundheitswesen als Fachkräfte des Gesundheitswesens definiert.

Im weiteren Verlauf der Analysen wurde die Gesamtstichprobe in 2 Gruppen unterteilt (Berufe mit und ohne Patient*innenkontakt), um mögliche Unterschiede der Belastungen dieser beiden Gruppen zu untersuchen. Folgende Daten wurden über die Online-Plattform SoSci Survey (Leiner 2019) erhoben:

- subjektiv empfundene Belastung durch COVID-19 (basierend auf den 8 Core-Items, zusätzlich einem weiteren Infektionsangst-Item von Frenkel et al. 2022 sowie 2 Items durch Erfahrungswerte aus Beratungskontexten; Tab. 1, Skala: *0*: *trifft gar nicht zu*; *6*: *trifft sehr zu*);
- Kenntnis, Inanspruchnahme und Bewertung möglicher innerbetrieblicher Hilfsangebote (selbstentwickelte Items; Tab. 2) sowie
- das psychosoziale Belastungserleben, operationalisiert als Depressivität durch eine deutschsprachige Kurzform des Patient Health Questionnaire (PHQ‑8; Kroenke und Spitzer 2002).

Zur Identifikation klinisch auffälliger Werte wurde der Cut-off-Wert ≥ 10 angewendet (Kroenke und Spitzer 2002; Kroenke et al. 2009). Zudem wurden soziodemografische Daten (Alter [in Kategorien] und Geschlecht) sowie die Berufsgruppenzugehörigkeit der Proband*innen erfasst.

**Table 1:** 

	Item
1.	Angst, andere zu infizieren
2.	Sorgen, die eigene Familie und Bezugspersonen mit COVID-19 anstecken zu können
3.	Reduzierte soziale Unterstützung infolge langer Arbeitszeiten und Stigmatisierung von Gesundheitsfachkräften
4.	Reduzierte Selbstfürsorge infolge von Zeit- und Energiemangel
5.	Flut von sich ständig ändernden Informationen
6.	Keine klaren Handlungsanweisungen/Vorgaben
7.	Gefühle von Isolation durch die Separation vom Team, mit dem Sie üblicherweise arbeiten
8.	Stigmatisierung Ihnen gegenüber bzw. gegenüber Personal, welches mit an COVID-19 erkrankten Patient*innen arbeitet
9.	Ressourcenknappheit (Personalknappheit, unzureichende Schutzkleidung, Tests etc.)
10.	Umgang mit einer Krankheit, zu der es noch kein Medikament gibt (Hilflosigkeit, neue Situation)
11.	Schwierige Vereinbarkeit von Beruf und Familie

**Table 2:** 

	Item	Antwortformat
1.	Haben Sie von den folgenden Angeboten des Universitätsklinikums schon einmal gehört?	Ja/nein
2.	Haben Sie eines oder mehrere Angebote des Universitätsklinikums schon einmal genutzt?	Ja/nein
	Falls ja, wie hilfreich fanden Sie dieses?	0: gar nicht hilfreich; 6: sehr hilfreich

Die Untersuchung beinhaltete zusätzlich 3 offene Fragen zu den tatsächlichen und präferierten Informationsquellen der Angebote und der favorisierten Art der Kontaktaufnahme (Tab. 3).

**Table 3:** 

	Item
1.	Woher haben Sie von den Angeboten erfahren?
2.	Wie würden Sie gern über solche Angebote informiert werden?
3.	Wenn Sie Beratung in einem oder mehreren der zuvor genannten Bereiche bräuchten, wie müsste die Kontaktaufnahme sein, damit Sie dafür ein Beratungsangebot gut annehmen könnten?

### Datenauswertung

Die Datenbereinigung umfasste die Ausschlüsse von insgesamt 237 Personen. Gründe für die Ausschlüsse waren, dass die entsprechenden Personen die Studie nicht vollständig ausgefüllt hatten (*n* = 218), > 25 % Missings in den zentralen Konstrukten (COVID-Belastungsscore, Depressivität) bzw. ≥ 20 % Missings insgesamt hatten (*n* = 6), den Fragebogen überdurchschnittlich schnell beantwortet hatten („careless responder“: *n* = 9) oder multivariate Ausreißer in den zentralen Konstrukten zu finden waren (*n* = 4). Nach der Datenbereinigung wies der Datensatz 3 fehlende Werte auf. Zusätzlich wurde eine einfache Imputation durchgeführt, um die fehlenden Werte der zentralen Konstrukte ergänzen zu können. Zur Überprüfung der Faktorenstruktur des COVID-Belastungsscore wurde zunächst eine konfirmatorische Faktorenanalyse durchgeführt. Die Berufsgruppenzugehörigkeit wurde in die Kategorien Beruf mit und Beruf ohne Kontakt zu Patient*innen zusammengefasst. Hierbei ist zu betonen, dass durch den Patient*innenkontakt kein automatischer Kontakt mit COVID-19-Patient*innen gegeben, das Risiko hierfür jedoch erhöht ist. Die 2 beobachteten Kriterien (COVID-Belastung und Depressivität) wurden in Form von multiplen Regressionen untersucht. Aufgrund der explorativen Natur der Analysen wurden alle prädeterminierten Prädiktoren in die initiale Berechnung eingeschlossen und anhand statistischer Parameter passende Modelle entwickelt. Stichprobenunterschiede wurden anhand zweiseitiger *t*-Tests für unabhängige Stichproben untersucht (mit vs. ohne Patient*innenkontakt). Die Effektstärke wurde mithilfe von Cohens *d* berechnet. Das Signifikanzniveau betrug bei allen Analysen α ≤ 0,05. Zur Überprüfung der Faktorenanalyse wurden 3 Modellgüteindizes mit unterschiedlichen Cut-off-Werten der Akzeptabilität verwendet (*Goodness of Fit Index* [GFI] ≥ 0,8; *Comparative Fit Index* [CFI] ≥ 0,8; *Root Mean Square Error of Approximation* [RMSEA] ≤ 0,08).

Die Freitextantworten wurden durch die Bildung von Kategorien qualitativ analysiert. Anhand von festgelegten Selektionskriterien wurden 30–50 % des Materials subsumiert und revidiert, das Gesamtmaterial kodiert sowie eine Häufigkeitsverteilung erstellt und interpretiert (Mayring 2015). Der Datensatz enthielt 547 Aussagen. Es bestand keine Verpflichtung, auf die offenen Fragen zu antworten. In die Auswertung eingeschlossen wurden jene, die auf mindestens eine der 3 offenen Fragen sinnvoll geantwortet haben.

## Ergebnisse

### Deskriptive Statistik

**Figure 1:**
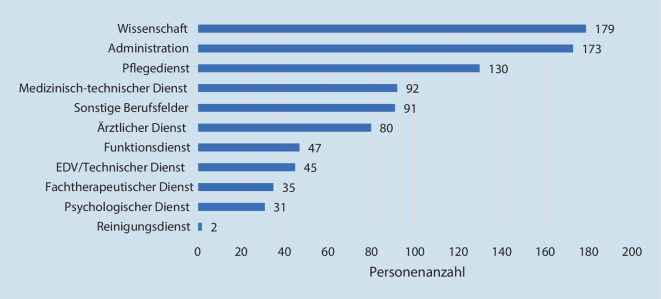

Die Verteilung der Berufsgruppen innerhalb der Stichprobe ist Abb. 1 zu entnehmen.

Der durchschnittliche Depressivitätswert (PHQ-8) betrug M = 7,40 (SD ± 5,13; Range: 0–24), wobei 267 Fachkräfte im Gesundheitswesen (31,08 %) einen klinisch auffälligen Wert (Cut-off ≥ 10) aufwiesen. Die Ausprägung des COVID-Belastungsscores betrug M = 21,85 (SD ± 10,95; Range: 0–54) sowie die der Infektionsangst M = 2,43 (SD ± 1,67; Range: 0–6). Die Teilnehmenden nahmen im Mittel 1,18 Hilfsangebote der Universitätsklinik in Anspruch (SD ± 1,94; Range: 0–7). Weitere deskriptive Kennwerte sind in Tab. 4 zusammengefasst.

**Table 4:** 

Versorgungsangebote	Kenntnis *n* (%)	Inanspruchnahme *n* (%)	Bewertung^a^ M (± SD)
Kurse, Seminare, Vorträge des betrieblichen Gesundheitsmanagements	643 (74,85)	260 (30,27)	4,23 (± 1,64)
Innerbetriebliche psychosoziale Beratung, Coaching, Supervision des betriebsärztlichen Dienstes	316 (36,79)	129 (15,02)	4,06 (± 1,44)
Betriebliche Sozial- und Suchtberatung	458 (53,32)	86 (10,01)	4,39 (± 1,65)
COVID-19: psychosoziales Beratungs- und Unterstützungsangebot	416 (48,43)	78 (9,08)	3,82 (± 1,75)
Beratung/Unterstützung in Führungs‑, Team- und Organisationsanliegen	206 (23,98)	133 (15,48)	3,71 (± 1,66)
Beratungsangebote des Personalrats	529 (61,58)	228 (26,54)	3,89 (± 1,75)
Klinikseelsorge	684 (79,63)	105 (12,22)	4,62 (± 1,32)

*n* = 859

*M* arithmetisches Mittel, *SD* Standardabweichung

^a^Antwortmöglichkeiten: 0 (gar nicht hilfreich) bis 6 (sehr hilfreich)

### Konfirmatorische Faktorenanalyse

Bei der konfirmatorischen Faktorenanalyse des COVID-Belastungsscores stellte sich heraus, dass 2 der ursprünglich integrierten Items (Tab. 1, Items 1 und 2) eine hohe Interkorrelation aufwiesen (*r* = 0,78). Zudem zeigte ein aus den beiden Items berechneter, gemittelter Gesamtscore nur eine geringe Ladung auf den Gesamtfaktor. Folglich wurde entschieden, den Summenscore der beiden Items aus dem finalen Modell (GFI = 0,90, CFI = 0,83, RMSEA = 0,129) zu entfernen und als weitere Variable (Infektionsangst) in die Regressionsmodelle aufzunehmen^1^.

### Ergebnisse der Regressionsmodelle

Die finalen Ergebnisse der 2 Regressionsmodelle sind in den Tab. 5 und 6 aufgeführt. Insbesondere zeigte sich ein starker signifikanter Zusammenhang zwischen dem Kontakt mit Patient*innen und der COVID-Belastung bzw. der Depressivität. Hinsichtlich der COVID-Belastung konnten zusätzlich Infektionsangst und Depressivität als Prädiktoren identifiziert werden. Die höhere COVID-Belastung, jüngeres Alter, weibliches Geschlecht und höhere Infektionsangst erwiesen sich als zusätzliche Prädiktoren von Depressivität.

**Table 5:** 

Variable	β‑Wert	Standardfehler	*t*-Wert	*p*-Wert
Intercept	14,03	0,70	19,93	< 0,001^***^
Kein Kontakt zu Patient*innen^a^	−5,54	0,62	−8,99	< 0,001^***^
Infektionsangst	1,44	0,18	8,02	< 0,001^***^
Depressivität	1,09	0,06	18,67	< 0,001^***^

*n* = 859, *R*^*2*^ *=* *0,41, F* *(3,855)* *=* *199,9, p* *<* *0,001*

^a^Variable wurde Dummy-kodiert

***p < 0,001, **p < 0,01, *p < 0,05

**Table 6:** 

Variable	β‑Wert	Standardfehler	t‑Wert	*p*-Wert
Intercept	1,42	0,57	2,48	0,01^**^
COVID-Belastung	0,27	0,01	18,85	< 0,001^***^
Alter	−0,21	0,06	−3,53	< 0,001^***^
Männliches Geschlecht^a,b^	−0,68	0,33	−2,06	0,04^*^
Diverses Geschlecht^a,b^	2,77	1,68	1,64	0,1
Kein Kontakt zu Patient*innen^a^	1,65	0,31	5,31	< 0,001^***^
Infektionsangst	0,19	0,09	2,06	0,04^*^

*n* = 859, *R*^*2*^ *=* *0,36, F* *(6,852)* *=* *81,25, p* *<* *0,001*

^a^Variable wurde Dummy-kodiert

^b^Variablen wurden in einer gemeinsamen Dummy-Variablen kodiert

***p < 0,001, **p < 0,01, *p < 0,05

### *t*-Test zu den Stichprobenunterschieden

Stichprobenunterschiede zeigten sich hinsichtlich der COVID-Belastung (*t* (857) = −5,89, *p* < 0,001, *d* = 0,43). Mitarbeiter*innen mit Kontakt zu Patient*innen hatten eine signifikant höhere COVID-Belastung als Mitarbeiter*innen ohne Kontakt. Die Analyse hinsichtlich Depressivität wurde nicht signifikant.

### Qualitative Analysen

**Figure 2:**
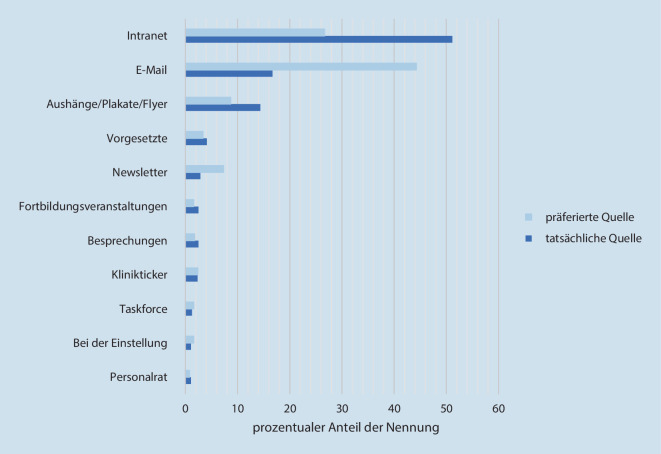

Die prozentualen Anteile der tatsächlichen bzw. präferierten Informationsquellen über die Hilfsangebote (Tab. 3, Items 1 und 2) zeigt Abb. 2. Die Teilnehmenden wünschten sich v. a. stärker durch E‑Mails (präferiert 44,27 % vs. tatsächlich 16,7 %) und Newsletters (präferiert 7,31 % vs. tatsächlich 2,87 %) informiert zu werden. Regelmäßige Informationen zu Beratungsangeboten per E‑Mail oder Newsletter sowie eine zentrale, prominente, einfache Angebotsdarstellung mit Kontaktadressen (26,15 %) wurden vom Personal gewünscht. Als favorisierte Kontaktmedien wurden E‑Mail (45,18 %) und Telefon (36,09 %) genannt. Eine persönliche Ansprache (7,16 %) sowie die Möglichkeit zur Online-Terminbuchung (11,57 %) favorisierten hingegen nur wenige Teilnehmenden. Zudem wünschte sich das Klinikpersonal eine unkomplizierte Kontaktaufnahme (27,69 %) mit zeitnaher Beantwortung/Terminvergabe (23,08 %) sowie einen vertraulichen, freundlichen bzw. urteilsfreien Rahmen (23,08 %). Als Kritikpunkte wurden die unübersichtliche Darstellung der Angebote und das erschwerte Auffinden von Informationen im Intranet/Internet erwähnt. Befürchtungen wurden hinsichtlich der Einsehbarkeit und Vertraulichkeit der Daten geäußert.

## Diskussion

In der vorliegenden Studie wurden anhand einer großen Stichprobe aus Fachkräften im Gesundheitswesen (*n* = 859) an einer Universitätsklinik der Grad des Belastungserlebens sowie Kenntnis, Inanspruchnahme und Bewertung innerbetrieblicher psychosozialer Versorgungsstrukturen untersucht. Weiterhin wurden qualitativ Informationen zu möglichen Anpassungen der existierenden Angebote erhoben.

### Belastungserleben bei Fachkräften im Gesundheitswesen

Die erhobenen Befunde (PHQ-8 ≥ 10: 31,08 %; M = 7,40) korrespondieren mit Vergleichsdaten von Fachkräften des Gesundheitswesens zu Beginn der Pandemie, wonach 18,90 % (Cao et al. 2020) bzw. 34,80 % (Chung und Yeung 2020) des Klinikpersonals einen klinisch auffälligen PHQ-9-Wert aufwiesen. Erhebungen während späterer Pandemiewellen fanden vergleichbare (PHQ-9 ≥ 10: 31,8 %; M = 7,74; König et al. 2021) bzw. noch höhere (PHQ-9 ≥ 10: 55 %; M = 9,08; Wild et al. 2022) Werte. Bei der Interpretation des Depressivitätswertes ist zu beachten, dass in der vorliegenden Studie der PHQ‑8 statt des PHQ‑9 verwendet wurde. Kroenke und Spitzer (2002) zeigten jedoch in ihrer Erhebung, dass beide Versionen ähnliche Likelihood Ratios hinsichtlich der Schweregradeinschätzung aufwiesen und somit gut miteinander vergleichbar sind. Angesichts (noch) fehlender Normwerte für ein COVID-spezifisches Belastungserleben beschränkt sich dessen Interpretation auf deskriptive Werte. Passend zu bereits bestehenden Befunden (Hill et al. 2022; Krishnamoorthy et al. 2020; Saragih et al. 2021) deuten beide Werte auf eine erhöhte mittlere Ausprägung hin. Als bedeutsamer Prädiktor sowohl für die Depressivität als auch für eine spezifische COVID-Belastung stellte sich der Kontakt mit Patient*innen heraus. Empirisch ist v. a. der Kontakt zu COVID-19-Patient*innen als Risikofaktor für die psychische Gesundheit von *frontline medical workers* gut dokumentiert (u. a. Cai et al. 2020; Hill et al. 2022; Muller et al. 2020). Ergänzend hierzu zeigen die Ergebnisse der vorliegenden Studie, dass auch Mitarbeiter*innen mit generellem Kontakt zu Patient*innen eine höhere COVID-Belastung aufwiesen als Mitarbeiter*innen ohne generellen Kontakt. Demzufolge könnte der generelle Kontakt zu Patient*innen bereits einen Risikofaktor für eine COVID-Belastung darstellen. Dies wird durch Daten chinesischer Studien gestützt, die bereits zu Beginn der COVID-19-Pandemie darauf hinwiesen, dass auch Personal, das nicht unmittelbar mit COVID-19-Patient*innen arbeitete, von psychosozialer Belastung betroffen war (Li et al. 2020; Liang et al. 2020). Zudem stellte sich sowohl hinsichtlich der COVID-Belastung als auch der Depressivität mehr Infektionsangst als ein bedeutsamer Prädiktor heraus. Bei der Beurteilung dieses Ergebnisses ist jedoch zu beachten, dass es sich um querschnittliche Befunde handelt und keine Kausalaussage getroffen werden kann. Dennoch stützt eine Vielzahl an Übersichtsarbeiten die Befunde, dass die Sorge um eine eigene Infektion sowie die Sorge, andere zu infizieren, ebenfalls massive Belastungen bei Fachkräften des Gesundheitswesens, die angesichts ihres Tätigkeitsfeldes teilweise einer höheren Exposition ausgesetzt sein können, auslösen können (u. a. De Kock et al. 2021; Hannemann et al. 2022; Muller et al. 2020). Des Weiteren zeigt sich in der vorliegenden Studie hinsichtlich der COVID-Belastung ein depressives Erleben als bedeutsam, zugleich erwies sich hinsichtlich der Depressivität eine spezifische COVID-Belastung als relevant. Diese Befunde deuten darauf hin, dass beide Konstrukte miteinander assoziiert sind und jeweils als Risikofaktoren füreinander fungieren: Eine COVID-spezifische Belastung könnte einen Risikofaktor für ein depressives Erleben darstellen sowie vice versa. Diese Befunde korrespondieren mit empirischen Studien, die psychisch belastete Personen als vulnerable Gruppen identifizieren, wonach die pandemiebedingten Stressoren einen Einfluss auf das Belastungserleben haben können (Manchia et al. 2022). Zudem passen diese Befunde zu einer Vielzahl von Studien, die eine erhöhte psychopathologische Belastung bei Fachkräften des Gesundheitswesens während der COVID-19-Pandemie zeigen (Umbrella-Review; Chutiyami et al. 2022). Zusätzlich erwiesen sich in den vorliegenden Analysen jüngeres Alter und weibliches Geschlecht hinsichtlich der Depressivität als signifikante Prädiktoren; diese sind empirisch ebenfalls bestätigt (weibliches Geschlecht [Morgan et al. 2022; Mulfinger et al. 2020] sowie jüngeres Alter [Bohlken et al. 2020; Manchia et al. 2022]). Empirische Befunde zeigen, dass während der Pandemie weibliche Beschäftigte im Gesundheitswesen höheren Risikofaktoren ausgesetzt gewesen zu sein scheinen. Hierzu zählen ein höheres Expositions- und Infektionsrisiko, ein erschwerter Zugang zu persönlicher Schutzausrüstung, eine höhere Arbeitsbelastung, weniger Führungs- und Entscheidungsmöglichkeiten, mehr Übernahme häuslicher Betreuungsaufgaben bei eingeschränkten Kinderbetreuungsmöglichkeiten sowie häufigere psychische Erkrankungen wie Depressionen, Angstzuständen und posttraumatischen Belastungsstörungen (Morgan et al. 2022). Auch ein niedrigeres Alter scheint einen Einfluss auf die psychosoziale Belastung zu haben, da jüngere Personen ebenfalls als Risikopopulation für psychische Beeinträchtigungen identifiziert wurden (Manchia et al. 2022; Rosales Vaca et al. 2022).

### Kenntnis, Inanspruchnahme und Bewertung innerbetrieblicher Versorgungsangebote

Bei mittlerer bis hoher Kenntnis der Angebote findet sich sowohl in der vorliegenden Untersuchung (9 %) als bei den oben geschilderten Implementierungserfahrungen eine vergleichsweise geringe Inanspruchnahme des spezifischen Beratungs- und Unterstützungsangebotes (Tab. 4). Passend hierzu verdeutlichen bisherige empirische Ergebnisse ein geringes Interesse an unternehmensinternen Hilfsangeboten (Muller et al. 2020) bzw. eine teilweise geringe Inanspruchnahme, wobei besonders Erholungsräume und kostenlose Mahlzeiten vom Personal geschätzt wurden (Branjerdporn et al. 2022). In der vorliegenden Studie beschrieb der Personalrat die höchste Inanspruchnahme (27 %), wobei hier vielmehr arbeitsrechtliche als psychosoziale Belange im Vordergrund stehen. Demnach wäre es möglich, dass das psychosoziale Angebot die wohl vorrangig arbeitsrechtlichen Beratungsbedürfnisse des Personals nicht bedienen kann. Die Zufriedenheit mit den wahrgenommenen Angeboten zeigte eine mittlere Ausprägung, wobei die Klinikseelsorge die beste Bewertung erhielt. In den bisherigen empirischen Studien zu Unterstützungsangeboten für Fachkräfte des Gesundheitswesens während der Pandemie wurden diese aufgrund ihrer zügigen Umsetzung nur wenig umfassend beschrieben oder evaluiert (Robins-Browne et al. 2022). Auch die Erhebung der Inanspruchnahme, der Bewertung des Angebots und die Untersuchung der Wirksamkeit der Maßnahme sind in vielen Studien bislang vernachlässigt worden (Branjerdporn et al. 2022; Robins-Browne et al. 2022) oder v. a. von deskriptiver Natur (Sheehan et al. 2022), sodass nur eine limitierte Einordnung der in der vorliegenden Studie erhobenen Befunde vorgenommen werden kann.

### Weiterentwicklung der Angebote

Die zur Kontaktaufnahme derzeit angebotenen Kanäle E‑Mail und Telefon wurden auch in der Umfrage als die präferierten Kontaktformen genannt. Im Gegensatz zur derzeitigen Darstellung der Angebote auf unterschiedlichen Homepages werden eine zentrale Angebotsdarstellung im Intranet sowie eine regelmäßige Zusendung der Angebote hauptsächlich per E‑Mails und Newsletters gewünscht. Diese Gegebenheiten und eine erwähnte Unübersichtlichkeit auf der Homepage der Uniklinik könnten Gründe für die geringe Kontaktaufnahme sein. Item 3 (Tab. 3) führte bei den Mitarbeiter*innen zu unterschiedlichen Antworten, Teile der Befragten antworteten explizit mit Medien, ein anderer Teil wiederum mit qualitativen Aspekten oder Verbesserungsvorschlägen zur Kontaktaufnahme, daher sollte bei weiteren Befragungen präziser formuliert werden, um eine noch zielgerichtetere qualitative Auswertung zu ermöglichen. Aus der Zusammenschau der bisherigen, teils empirischen Literatur und der eigenen Implementierungserfahrungen erscheint ein auf die Bedürfnisse der Fachkräfte angepasster *stepped care approach* sinnvoll, wonach für eine kleinere Anzahl von Mitarbeitenden, bei denen das Risiko einer psychischen Erkrankung besteht oder die bereits psychisch erkrankt sind, ein ihrer Belastung entsprechendes Hilfsangebot vorgehalten werden sollte (Branjerdporn et al. 2022; David et al. 2022; Robins-Browne et al. 2022). Insgesamt erscheinen innerbetriebliche Angebote für das Klinikpersonal sinnvoll und gewünscht, wenn sie leicht erreichbar und vertraulich sind.

### Stärken und Limitationen der Studie

Wesentliche Stärken der Studie sind zum einen der multimethodale Aufbau mit der Erfassung sowohl quantitativer als auch qualitativer Daten, die sich auf eine überzeugende Klinikstichprobe von *n* = 859 stützen. Weiterhin hervorzuheben ist die differenzierte Erfassung von Kenntnis, Inanspruchnahme und Bewertung von innerbetrieblichen Unterstützungsangeboten. Wenngleich die Generalisierbarkeit der Ergebnisse durch die Beschränkung auf eine Universitätsklinik teils eingeschränkt erscheint und zudem ein Selektionsbias bezüglich der Stichprobe vorliegen könnte, können die Ergebnisse einen Beitrag zu Konzeptualisierung, Implementierung sowie Bewertung von innerbetrieblichen Unterstützungsstrukturen für Fachkräfte im Gesundheitsweisen leisten. Weiterhin ist die differenzierte Untersuchung von sowohl COVID-Belastung als auch einer besonders häufig vorkommenden psychopathologischen Störung (Depressivität) zu nennen. Doch um die Erhebung möglichst ökonomisch zu gestalten sowie aus datenschutzbezogenen Gründen, wurde auf den Einbezug einiger Zusatzaspekte verzichtet. Weder das Aufsuchen externer Hilfsangebote noch Häufigkeit, Regelmäßigkeit, Zeitraum und Grund (COVID-Belastung) der Inanspruchnahme unternehmensinterner Hilfsangebote wurden kontrolliert. Diese Aspekte hätten jedoch Auswirkungen auf die Interpretation der deskriptiven Daten. Zudem ist der COVID-Belastungsscore aufgrund seiner Aktualität noch nicht validiert worden, und die Ergebnisse sind demzufolge vor diesem Hintergrund zu interpretieren. Zuletzt ist das querschnittliche Studiendesign der Untersuchung als limitierend zu erwähnen; dieses lässt ausschließlich die Interpretation korrelativer Zusammenhänge zu und erlaubt keinerlei kausale Schlussfolgerungen.

## Fazit für die Praxis

Aus den vorgestellten Ergebnissen lassen sich folgende Schlüsse und Implikationen ziehen:

- Berufsgruppen mit direktem Kontakt zu Patient*innen weisen eine besonders hohe COVID-Belastung auf.
- Trotz der hohen psychosozialen und pandemiebedingten Belastung werden innerbetriebliche Angebote nicht stark frequentiert.
- Als Verbesserungsmaßnahmen der Hilfsangebote wünscht sich das Klinikpersonal die Ausweitung der Kontaktaufnahme auf E‑Mail und Telefon sowie eine einfache, zentrale und übersichtliche Darstellung der Beratungsangebote (einschließlich Kontaktadressen) im Intranet.
- Innerbetriebliche Versorgungsangebote für Klinikpersonal sollten leicht und schnell erreichbar sein und ihre Datenschutzmaßnahmen offenlegen.

## Footnotes

1 Nähere Informationen zum Messmodell können bei den Autor*innen erfragt werden.
